# Curiosities of American Lunacy

**Published:** 1894-08-25

**Authors:** 


### CURIOSITIES OF AMERICAN LUNACY.

"A Medical Superintendent" writes: Under your annotation, "Curiosities of American Lunacy," you refer to various novel delusions observed by Dr. Brown of Chicago. I would put in a plea for the observation, recording and classification of delusions with reference to the medical aspects of each case. A great mass of undigested information would be so dealt with. The delusions of the insane are usually confined within a narrow compass—queens, unpardonable sinners, magnates and millionaires abound in every asylum, and there seems to be little originality in the invention of new delusions, while each patient is limited to his own particular ideas. The inception, development, and progression of fixed delusions ought always to be obviated by every possible means. They are of passing interest to asylum nurses. At first the strange topsy-turveydom of the insane life is curiously watched, but it requires constant and enthusiastic attention to maintain the needful interest. When strange and unusual forms of delusion are presented they cannot fail to be noticed, but the common aberrations are quite as important from the medical point of view. We have lately had two cases who habitually mistook their reflections in mirrors for other people, and argued with their own features in every variety of mood. Another strange case is that of a young lady who complains that her thoughts drop out of her nose and fall tinkling on the floor. When remonstrated with for littering the carpet with her thoughts, and her nurse was told to sweep them up, she at once found fault with the "stupidity" of such an order because of the impossibility of sweeping up thoughts!

